# Zebrafish tracking using convolutional neural networks

**DOI:** 10.1038/srep42815

**Published:** 2017-02-17

**Authors:** Zhiping XU, Xi En Cheng

**Affiliations:** 1 School of Computer Science, Shanghai Key Laboratory of Intelligent Information Processing, Fudan University, Shanghai, China; 2 Jingdezhen Ceramic Institute, Jingdezhen, China

## Abstract

Keeping identity for a long term after occlusion is still an open problem in the video tracking of zebrafish-like model animals, and accurate animal trajectories are the foundation of behaviour analysis. We utilize the highly accurate object recognition capability of a convolutional neural network (CNN) to distinguish fish of the same congener, even though these animals are indistinguishable to the human eye. We used data augmentation and an iterative CNN training method to optimize the accuracy for our classification task, achieving surprisingly accurate trajectories of zebrafish of different size and age zebrafish groups over different time spans. This work will make further behaviour analysis more reliable.

Computer-aided tracking of multiple same-size zebrafish with maintained identity after occlusion is still an unresolved problem in ethology^1^. State-of-the-art technologies cannot keep correct identities for long term after severe occlusion when tracking a large number of objects. Tracking multiple zebrafish with identity maintained correctly over the whole video is critical for individual behaviour analysis. When more than two fish interact with each other physically or swim across each other, these fish will enter a state of occlusion in the top camera’s view (as illustrated in Fig. 1).

After occlusion happens, three possible situations may occur. In the first situation, the identity of the fish is not maintained after occlusion, and a new identity is generated instead. In the second situation, identities are linked across occlusions based on the individual movement prediction mechanism such as Kalman filter^2^ or particle filter^3^ -based prediction framework. If occlusions are too complex to link identities after occlusion, then the first situation happens. Manual review and correction should be made at this point. In the third situation, the trajectory still belongs to the original identity before occlusion without errors or miss-assignments of the identity. Artificial marking is a way to solve this problem, but in some cases, this solution is not plausible when the population is big or an individual fish is too small. Some general traits such as size and shape can identify individuals when individuals vary greatly in size or shape or at low densities. Fingerprinting^4^, like features, is used to recognize individuals, but this method is currently limited to small fish groups (≤20 fish) or time spans longer than 5 min in order to acquire a video with a portion with no crossings that is long enough for its frames to be used as reference images. Sometimes, when the crossings are frequent or the time span of the recorded video is less than 5 min, this mechanism will always fail. From the above, we can see that the identity switching and lost identification problem cannot be solved completely with state-of-the-art tracking methods after more than two fish have been occluded when the number of fish is big. Moreover, identity error is not a local error, and this error will propagate through the remaining image sequence. This error will make the individual tracking data as well as further individual behaviour analysis unusable.

A new type of learning mechanism, namely deep learning, especially the convolutional neural network (CNN)^5^, shows the ability to distinguish human faces directly with very low error rate^6^. A CNN also has the ability to track one object in a complex environment with higher accuracy than traditional methods. The deep learning tracker (DLT)^7^, which is based on a stacked denoising autoencoder network, was the first tracker based on CNN tracking methods reported in the literature. Some researchers use an ensemble CNN^8^ and maintain a pool of CNNs to track an object, but a CNN needs sufficient training data, which these methods cannot provide in advance, so these methods only show comparable results against other state-of-the-art trackers. Fan *et al*.^9^ proposed a human tracking method based on CNN. Chen *et al*.^10^ constructed an effective CNN-based appearance model to track an identifiable object. CNNs show great potential in object tracking and object recognition; however, to our knowledge, they have not been applied to tracking animals of the same species with almost the same size and length, especially if they are indistinguishable to the human eye.

We have developed CNNTracker, a software application that tracks each fish in a group and maintains the correct identities for different fish numbers and time spans. As illustrated in Fig. 2, CNNTracker first extracts head feature maps from each frame for each individual fish. These head feature maps are used to identify individuals in each frame, maintaining the correct identities even after crossings or occlusions. The head point pairs between two successive frames are formed using the displacement between the same head points across two frames and head feature maps of the same fish. Segments of the fish trajectories can be obtained by linking the corresponding head point pairs. The initial trajectories for training are generated based on the displacement between segment end and start points and the frame difference between the end and start points. Some segments may share the same time stamp over a short time span; if the number of these segments is same as the total number of fish in the image sequence, then these segments can be fused with the initial trajectories and can be regarded as an initial training sample for CNN training. By using an iterative CNN training method to optimize the accuracy of the CNN, each segment of the trajectories is fed into the final trained CNN to determine to which identity it belongs. These segments are linked according to their assigned identities to form trajectories in temporal order. Finally, the software detects and corrects trajectory errors, fills trajectory gaps, and evaluates the credibility of the trajectories. The whole process is fully automatic and does not suffer from the propagation of errors, giving reliably correct identities for any complexity of crossings. None of our methods are specific to zebrafish tracking, and the same approach should be readily applicable to tracking tasks for other species of fish. It is easy to use, needing no modifications for different species of fish (only five parameters must be input by the user; see Supplementary Note).

## Results

We used five typical video sequences to run benchmarks on the tracking system. The first data set D1 (see Video S1), captured at 60 frames per second, recorded 14 zebrafish swimming in a tank and contains 2,000 frames at a resolution of 2,048 × 2,040. The second data set D2 (see Video S2), captured at 50 frames per second, recorded 25 wild-type zebrafish swimming in the same tank and contains 5,000 frames at a resolution of 2,048 × 2,040. The third data set D3 was captured at 30 frames per second, consists of five wild-type zebrafish swimming, contains 15,000 frames at a resolution of 1528 × 1080. The fourth data set was captured at 50 frames per second and is an example video of five zebrafish provided as a courtesy from http://www.idtracker.es/. The fourth data set D4 (see Video S3) recorded 11 wild-type zebrafish swimming in a tank and contains 4,000 frames at a resolution of 2048 × 2040. The fifth data set D5 (see S4 Video) contains two parts recorded at different times (day 1 (P1) and day 3 (P2)) and recorded 25 wild-type zebrafish swimming in the same tank. The first part contains 10,000 frames and the second part contains 11,000 frames, both at a resolution of 2048 × 2040.

To quantitatively evaluate the proposed tracking software, its tracking performance was compared with ground truth data. We used the seven metrics shown in Table 1 to evaluate the performance of the proposed system against ground truth data. The result of the performance evaluation based on the five typical video sequences is listed in Table 2. A correctly tracked object is defined such that the distance between the *x* and *y* coordinates of tracked object and *x* and *y* coordinates of the ground truth object is less than 70 pixels in the same frame.

As shown in Table 2, the proposed method performs significantly well on all metrics. It shows that the CNN-based tracking system is robust when tracking a large group of the same fish species. Even for D5, the automatically trained CNN in one experiment (P1) could be used for several days, allowing the identification of individuals across different videos (P2). Unlike the fingerprint mechanism used in ‘idTracker’^4^, the proposed method only used the head region as the feature sampling region instead of the whole body because the head region of fish is relatively stable and the normalized head region feature map can act as the ‘identification photo’ of a fish.

### Fish head feature map generation

**Figure 3:**
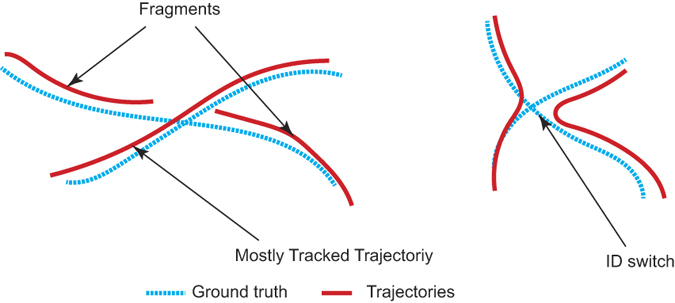
Tracking evaluation criteria. The blue dotted lines are ground truth trajectories and red lines are the trajectories generated by the tracking algorithms.

Fish head image generation is an essential part of the proposed tracking system. From the top view of the fish image, the intensity values of the fish head region are darker than the water, and the head region is partially elliptical (as illustrated in Fig. 3). Based on these characteristics, the head region can be regarded as a blob.

Suppose a pixel point is denoted as (*x, y, σ*) in scale-space, where *x* and *y* are the point’s coordinates and is the scale of the point. The Hessian matrix of this point is defined as:


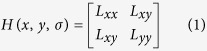


where *L*_*xx*_, *L*_*xy*_ and *L*_*yy*_ are the convolution of the second order Gaussian derivatives with the 2D image *I* at point (*x, y*) at scale *σ*. The scale-space can be obtained by convolving the image with a Gaussian kernel function at different scales *σ*. The *doH* (Determinant of Hessian) of matrix *H* can be expressed as:


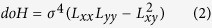


where *L*_*xx*_, *L*_*xy*_ and *L*_*yy*_ are the convolution of the second order Gaussian derivatives with the 2D image *I* at point (*x, y*) at scale *σ*. The blob points 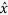, 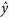 and scales 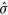 can be derived from


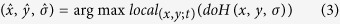


**Figure 4:**
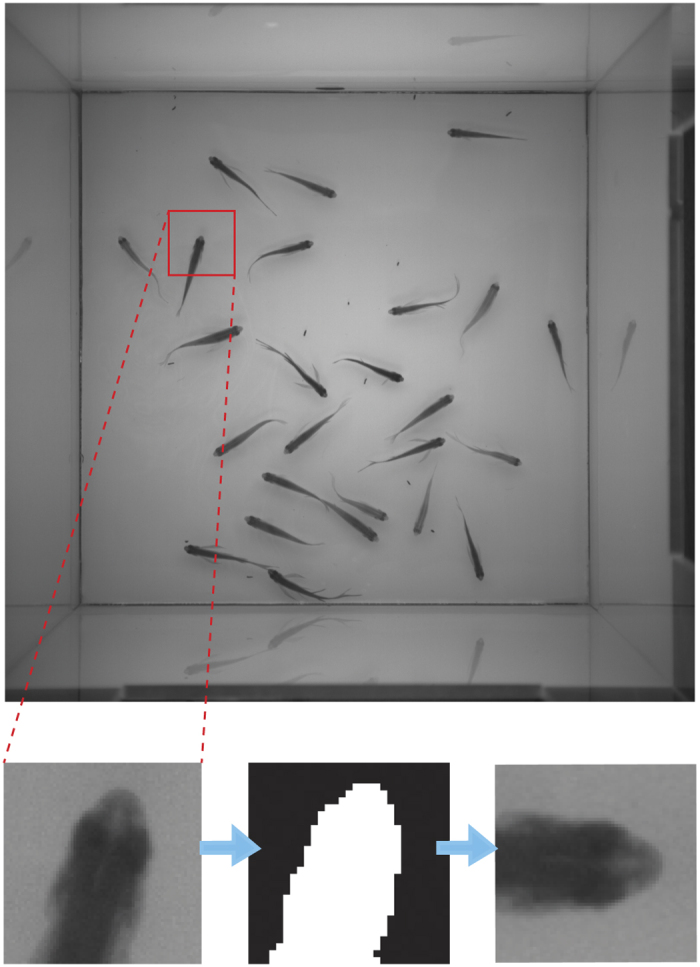
Fish head feature map generation. The centre of the red square is the detected fish head. We extract the image patch with the head point as the centre of the dashed red line illustrates, and we transform the head patch into a binary bitmap, extract the coordinates of the white pixels in the binary image, and use PCA to obtain the orientation of the fish head. We then rotate the patch to make the fish head point to the right at 0° as the final fish head feature map.

Blob points not only exist in the fish body regions but also occur in the non-fish body region. These non-fish body points not in the fish body regions should be filtered out according to the specified scale range and the intensity range of the head point. With the head point as the centre, we obtain a square patch cantered on the fish head (as illustrated in Fig. 4). The intensities of the fish head pixels have strong contrast with the background, so we use Otsu’s method^11^ to obtain the binary version of the head (as illustrated in Fig. 4). After the orientation of the head is obtained by Principal Component Analysis (PCA) based on the coordinates of a binarised patch bitmap, all head patches are rotated to a 0° head orientation to the right as the head feature map (as illustrated in Fig. 4).

### Inter-frame feature matching

Inter-frame feature matching is based on a comparison between the binary version of the head feature map of the current frame and its counterpart in the previous frame. The current frame’s candidate head point counterpart in the previous frame will be located near the position of the previous head point position in the current frame because the swimming displacement of the fish is limited in consecutive frames. The binarised head feature map *B*_*t*_ of a candidate’s head points in the current frame is compared with its counterpart *B*_*t*−1_ in the previous frame according to the following equation,


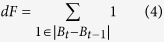


where *B*_*t*−1_ is the binary version of head feature map in the previous frame, *B*_*t*_ is the binary version of head feature map in the current frame. Then, from the candidate points in the current frame, we aim to identify the head point with the smallest difference *dF*dF in the previous frame to form a pair of matching head points. After gathering all pairs of head points, we retain the pair with the smallest *dF* between the previous and current head orientations.

### Trajectory segment formation

The complete sequence of captured frames is composed of a series of pairs denoted by set {*U*_*i*_}, where *U*_*i*_ is the two columned head point pairing list between successive frames. The first column contains a fish head point in the previous frame, the second column contains the counterpart head point in the current frame, and *i* is the frame number. We then connect the matched pairs in {*U*_*i*_} to form the trajectory segment. First, we locate the first available matching pair 〈*a, b*〉 in *U*_*i*_ as the initial portion of trajectory segment *S*, and delete this item in *U*_*i*_. We then use the second item *b* in 〈*a, b*〉 as the search item in the first column of *U*_*i*+1_ according to the order of captured frames. If the first column of *U*_*i*+1_ contains an item such as b, then we append 〈*b, c*〉 onto the current trajectory segment *S* and delete this item in *U*_*i*+1_. If the first column of *U*_*i*+1_ does not contain item *b*, then we end current trajectory segment *S*. We then begin to locate another first available matching pair 〈*d, e*〉 in {*U*_*i*_} as the initial portion of new trajectory segment *S*′. After obtaining all trajectory segments, we delete too-short segments which contain frame numbers less than *η*.

### Automatic CNN training and training data generation

**Figure 5:**
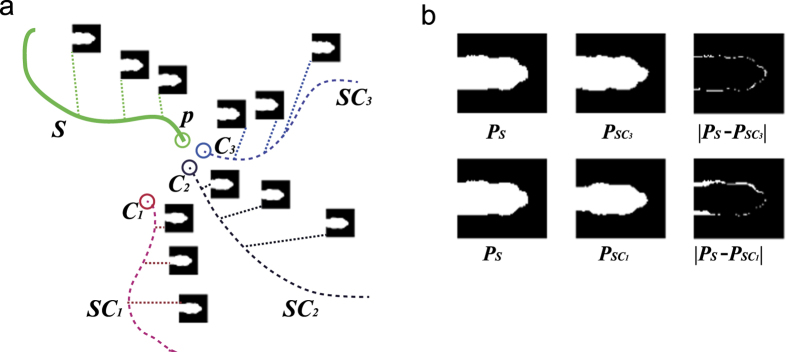
Initial trajectory generation and binary version of the head feature map comparison. (**a**) *S* is the base part of the trajectory (green line) and the green circle indicates its end point *p. SC*_1_, *SC*_2_ and *SC*_3_are three linkable candidate segments that satisfy the specified spatial-temporal conditions. (**b**) Comparison of the binary version of the head feature maps in *S* with their counterparts in *SC*_1_ and *SC*_3_.

The initial training data is generated based on two facts: each segment only belongs to one identity and the total number of identities is known in advance. As illustrated in Fig. 5(a), we take the first unused trajectory segment *S* as the base part of one trajectory, find candidate trajectory segments (like *SC*_1_, *SC*_2_ and *SC*_3_in Fig. 5(a)) that i) have a start point within a circle cantered at the end point of the segment (the green circle in Fig. 5(a)) with a radius of 600 and ii) the candidate segment start point is less than 200 frames from the end point of segment *S*. If there is only one trajectory segment that starts within 90 pixels and 10 frames of the end point of, this trajectory segment can be regarded as the next base part of the trajectory. Otherwise, we compare the binary version of head feature maps in *S* (as illustrated in Fig. 5(b)) with those of the candidate segments (like *SC*_1_, *SC*_2_ and *SC*_3_ in Fig. 5(a)) using Eq. 4 to find the segment with the smallest *dF* as the next base part of the trajectory. Further, *dF* must be below a certain value (in our experiment, this value is 100). We continue this process until there are no more candidate segments, then we select another unused trajectory segment as the base part of another trajectory, and repeat the above-mentioned process until the total number of trajectories is the same as the total number of fish in the video sequence. We then remove all the duplicate segment IDs in the trajectories because each segment only belongs to one identity.

**Figure 6:**
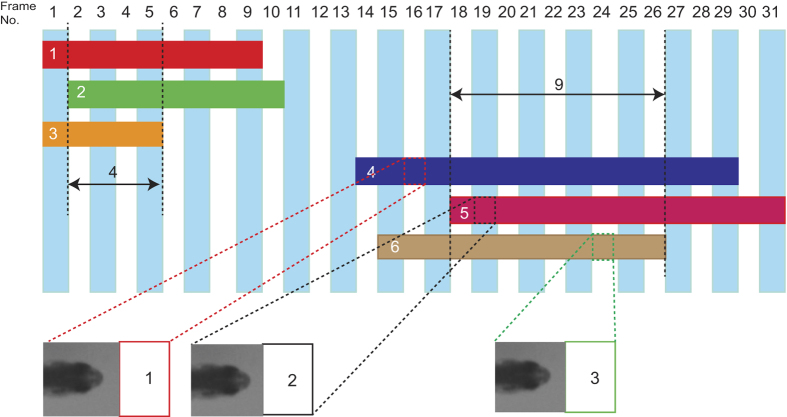
Segments sharing frame numbers. Here, there are three identities in the video sequence and six segments in frames 1 to 31. Segments 1, 2, and 3 form one collection, and segments 4, 5, and 6 form another collection. Segments 4, 5, and 6, which have the most shared frame numbers, can act as the initial training dataset.

Some segments may share the same frame number. As illustrated in Fig. 6, assume there are three identities recorded in the whole video sequence. Segments 1, 2, and 3 shares four frames in common and Segments 4, 5, and 6 share nine frames in common. Segments that share several frames are called a collection. Segments 1, 2, and 3 are a collection, and segments 4, 5, and 6 also are a collection. The segments in adjacent collections in a time span can be linked with each other based on the smallest *dF*, as mentioned above. The binary version of the head feature maps in the segment in one collection are compared with the binary version of the head feature maps in the segments in another collection using Eq. 4 to find the segment ID pairs with the smallest *dF*. The segment ID pairs can be added to the above generated initial trajectories.

The head feature maps stored in the segment in the trajectory along with the trajectory ID form the initial training dataset. The initial dataset may be very small, so we use data augmentation to increase the training data set by shifting the head point by ±1 pixels in the horizontal and vertical directions and generate new head feature map. This newly generated head feature map with the original identity is added to the initial training dataset.

The training dataset is divided into two parts, the training part and test part. These two parts correspond to two different modes of the CNN: training and test. The head feature maps in the test part are used to measure the error rate of the CNN. In training mode, the CNN is repeatedly given fish head feature maps with the correct ID (label) so that it can learn to classify each fish. In test mode, the CNN’s performance is evaluated on a set of different fish head feature map with the learning mode offline.

### Architecture of the proposed CNN

**Figure 7:**
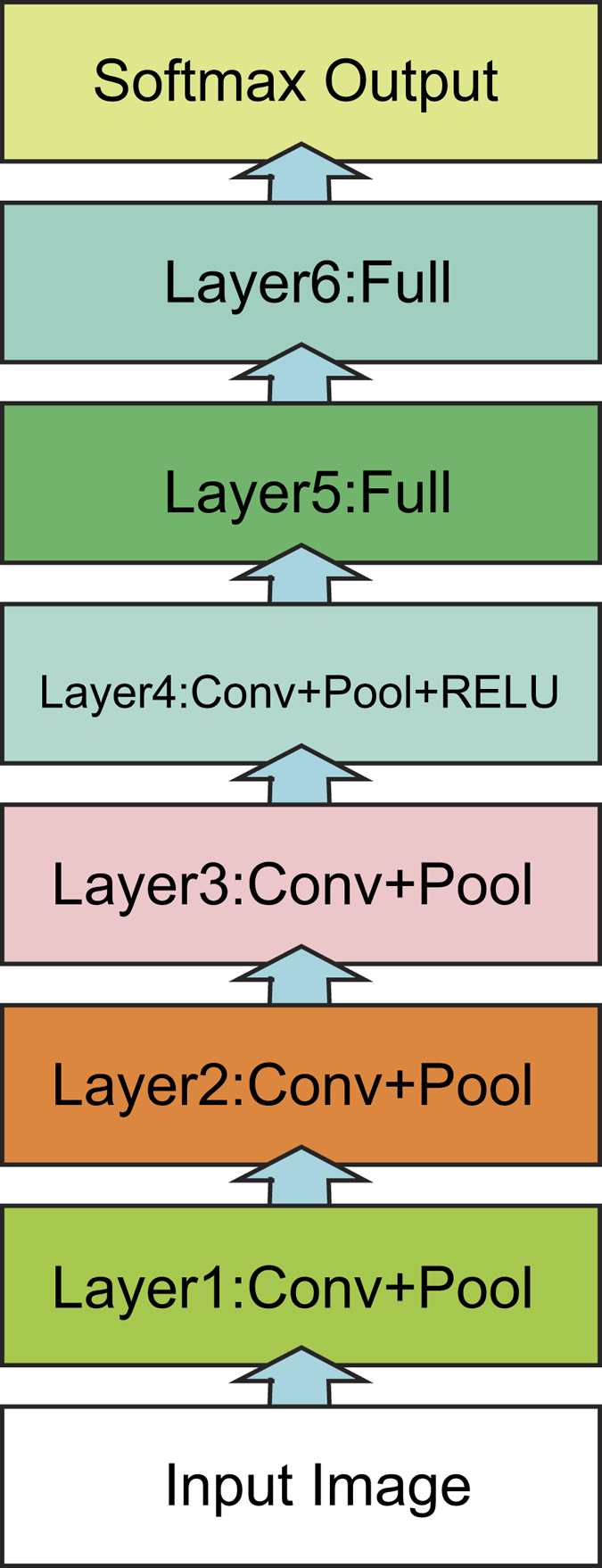
Structure of the CNN.

The CNN in the proposed method is composed of convolution, pooling, rectified linear unit (ReLU), and dropout layers. In the convolution layer, the weights of each convolution kernel are trained by the backpropagation algorithm. Each layer contains many convolution kernels, and each kernel is shared over the entire image with the same parameters. The convolution operators can extract different features from the input layer. The ReLU is a layer of neurons that use the non-saturating activation function *f*(*x*) = *max*(0, *x*). It not only increases the nonlinear properties of the decision function and of the overall network without affecting the receptive fields of the convolution layer but also makes the training speed several times faster. Pooling performs non-linear down-sampling on the input image, splits the image into a set of non-overlapping regions and, for each such sub-region, outputs the maximum or average. The pooling layer provides a form of translation invariance. A dropout layer randomly sets the layer’s input elements to zero with a given probability, which was set to 0.2 in the proposed method. This corresponds to temporarily dropping a randomly chosen unit and connections from the network during training. The dropout layer might help prevent overfitting. The architecture of the CNN used in this research study is shown in Fig. 7.

For each layer of the network, the specific configurations of the network parameters are shown in Table 3. The input layer is the input image layer: each input image is scaled to 65 × 65 and is a grayscale image. Layers 1 to 4 are convolutional layers. Taking Layer 1 as an example, the convolution filter size is a block of 3 × 3, the convolution stride is 1, and there is a total of 30 channel convolution filters. In Layers 1, 2, and 3, after the convolution filtering, they are also connected with a max-pooling operation with a pool size of 2, 0 padding, and stride of 2. In Layer 4, after the convolution filtering, it is also connected with a max-pooling operation and ReLU operation layer. Layers 5–6 are fully connected layers. Taking Layer 5 as an example, the number of neurons in this layer is set to be four times *num*, the total number of fish. The number of neurons in Layer 6 is equal to *num*.

We use MatCovNet^12^ as the base for implementing our CNN architecture. During the training process, a set of head feature maps with ID labels were fed into CNN multiple times and CNN used a stochastic gradient descent to tune the weights to learn from the labelled data. A training epoch is finished when all head feature maps in the training set have been presented to the CNN. The training process was done on a GTX 980Ti for 30 epochs using the initial training set.

The trained CNN was then applied to the remaining segment collections. As mentioned above, each collection has the same number of segments as the total number of IDs. Each segment only belongs to one identity. The trained CNN used each head feature map stored in the segment to determine which ID it belongs to. We then counted the frequency of each ID in the segment and chose the ID that had a frequency of over 0.2 as the candidate ID of the trajectory segment. Because the initial training dataset is small, the performance of the initial version of the CNN is not stable, and it will cause multiple IDs to be assigned to the same segment.

To fix this problem, we need to extend the training set. As mentioned before, a collection has the same number of segments as the total IDs in the image sequence. We keep all segments with one identity assigned in a collection and record these identities as *ID*_*true*_, remove IDs containing *ID*_*true*_ from the multi-ID assigned segments, then collect all segments that have one assigned ID. If the total number of segments with one assigned ID is just same as the number of fish in the video sequence, then these segments can be added to the initial training data set. After the remaining collections have been processed like this, an extended version of the training set will be generated. The extended training data set will be fed into the CNN for re-training. This process is applied to all the remaining collections until the number of collections added to the training dataset is more than half of the total number of collections, and a final trained version of CNN is obtained.

This CNN is then applied to the head feature maps contained in the trajectory segments not in the training dataset. Each head feature map is fed into the CNN, and CNN assigns a fish ID to this image. The segments then have a list of fish IDs; we then count the frequency of each ID in the segment and chose the ID that has a frequency of over 0.2 as the candidate ID of the trajectory segment. We then filter out short segments (containing less than 10 frames) assigned with three or more identities to ensure it will not disturb the next segment linkage step.

### Linking trajectory segments to form trajectories

After each trajectory segment is automatically assigned an ID via the CNN, the segment with an assigned ID containing the smallest frame number acts as the initial part of the trajectory. We then choose the end coordinates of the base segment as the base point and, select all segments with the same ID that begin within 1,000 frames and start within of 2,000 pixels as the candidate segments for the link with the base segment. Among these candidates, we next choose the segment with the smallest starting frame number as the new base segment and link this segment to the old base segment until there are no more segments available. When there are no more segments available, we choose another segment with a different ID as the initial part of another trajectory and run the above process again until all IDs have been processed.

### Error detection and correction

Each trajectory segment can correspond to only one trajectory. If a trajectory segment occurs in multiple trajectories, an error has occurred and should be corrected. Errors can be easily identified by summing the number of occurrences of the trajectory segments over all trajectories. If this sum is greater than one, an error has occurred. The detected error segment may occur in more than two trajectories. We locate segments before error segment *seg*_*err*_ in one trajectory and extract coordinate *p*_*i*_ and frame number *n* at the end of the located segment. Starting from frame *n* to *n* + 1, we use the Farneback^13^ method to calculate the optical flow of each pixel between these two frames, and then use the magnitude of optical flow bigger than one pixel in the surrounding *w* pixels of *p*_*i*_ as the selection mask *m*. We use a ten-bin histogram to find which orientation range [*θ* − *b*/2, *θ* + *b*/2] of the optical flow in mask *m* is the major flow, where *b* is bin width. After the major orientation *θ* is obtained, with the head point as the centre and the positive direction of the X-axis as 0 degrees, we draw ray segments with orientation *θ* from *p*_*i*_ with length *w*. With equidistant sampling *w* points on the ray segment, we obtain maximum magnitude *ρ* from the segment and move *p*_*i*_ to a new position (*p*_*x*_ + *ρ*cos(*θ*), *p*_*y*_ + *ρ*sin(*θ*)). We then move to frame *n* + 1, and perform the above-mentioned process again until we reach the frame number of the start point of error segment *seg*_*err*_, and calculate distance *dis*_*i*_ between the coordinate *p*_*err*_ and *p*_*i*_. We then perform the above-mentioned process on another trajectory with error segment *seg* and get another distance *dis*_*i*+1_ between the coordinate *p*_*err*_ and pi + 1 *p*_*i*+1_ until all trajectories containing error segments are processed. Then, segment *seg* in the trajectory with minimum distance *dis*, where is below a specified threshold, can be regarded as the correct assigned *seg*. We then delete other segments *seg* in other trajectories.

### Gap filling and trajectory verification

After all trajectories have been generated, some trajectories may contain time series gaps (discontinuous frame numbers caused by occlusion). When one trajectory is selected, the start *N*_*gapstart*_ and end *N*_*gapend*_ frame number of the gap in this trajectory can easily be identified, and we then extract position *p*_*start*_(*x*_*start*_, *y*_*start*_) and *p*_*end*_(*x*_*end*_, *y*_*end*_) from the trajectory from frames *gap*_*start*_ and *gap*_*end*_. Starting from frames *N*_*start*_ to *N*_*start*+1_ and assigned *p* with *p*_*start*_(*x*_*start*_, *y*_*start*_), we again use the Farneback method to calculate the optical flow of each pixel between two frames, we then select the magnitude of optical flow bigger than one pixel in the surrounding *w* pixels of *p* as the selection mask m. We use a ten-bin histogram to find out which orientation range [*θ* − *b*/2, *θ* + *b*/2] of optical flow in mask *m* is the major flow, where *b* is bin width. We then obtain the ratio *r* of orientation in mask *m* and record *r* in list *l*_*r*_. After major orientation *θ* is obtained, with the head point as the centre, and the positive direction of the X-axis as 0 degrees, we draw ray segments with orientation *θ* from *p*_*i*_ pi with length w. With equidistant sampling of *w* points on the ray segment, we obtain the maximum magnitude *ρ* from the segment, move *p* to a new position (*p*_*x*_ + *ρ*cos(*θ*), *p*_*y*_ + *ρ*sin(*θ*)), and record *p* in list *l*_*p*_. We then move to frames *n* + 1 to *n* + 2 and repeat the above-mentioned process again until we reach frame number *N*_*end*_. We then calculate the portion *p*_*r*_ of the items in *l*_*r*_ that are greater than 0.5. If the distance between *p* and *p*_*end*_(*x*_*end*_, *y*_*end*_) is less than 20 pixels, we use list *l*_*p*_ to fill the gap. Otherwise, if the distance between *p* and *p*_*end*_(*x*_*end*_, *y*_*end*_) is greater than 300 pixels and *p*_*r*_ is greater than 0.6, we remove the segment with start frame number *N*_*end*_ and mark this trajectory for further processing. If neither of the above cases is true, we linearly fill the gap from its beginning to its end. We then redo the above-mentioned gap filling process on the marked trajectories.

We then calculate the displacement of each fish between frames. If the displacement is too high above a threshold *ξ*, we regard the trajectory at that frame to contain an error. We then locate the segment which causes this error, delete it from the trajectory, and apply the above mentioned gap filling process to this trajectory to obtain the final error-free trajectory.

## Discussion

The proposed system utilizes the fact that animals of the same congener can be identified individually^14^,^15^,^16^. Previous tracking methods using Kalman or particle filters often lose or incorrectly assign IDs after tracked objects overlap in an image. In our system, as long as the fish head is not occluded, the head point can still be detected; thus, it outperforms blob-based tracking systems. The head is more rigid than other parts of the fish, and the variations in the image texture and signature are minor. ‘idTracker’ perceives tracked objects as blobs, and it uses an entire blob as the feature base, which will cause feature instability. A CNN can be utilized to classify fish, even they look similar to the human eye. We take a fish head feature map from the fish head region as the biometrics of a fish, just as in human face recognition. Data augmentation enhances the performance of CNN when CNN face small training data set.

**Figure 8:**
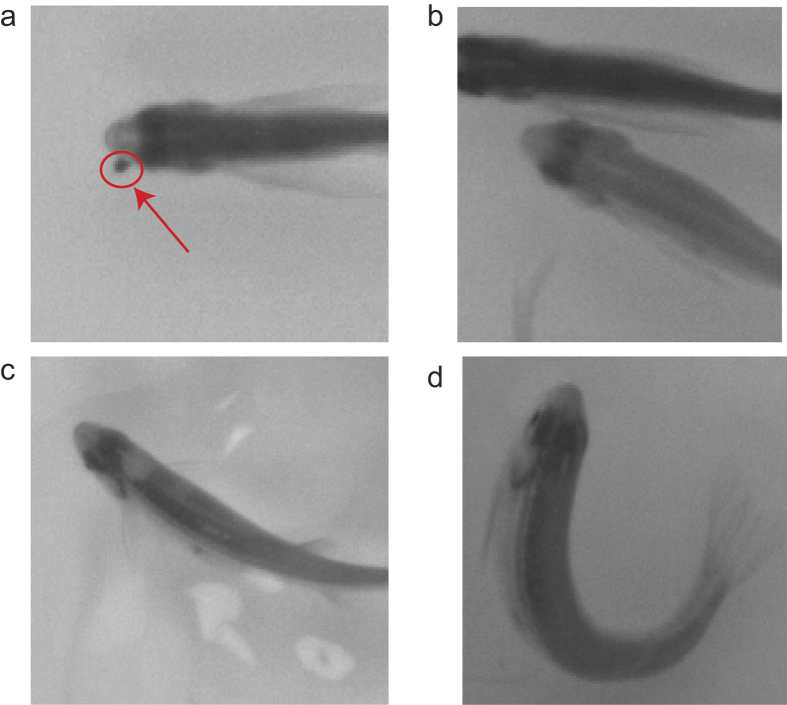
Some cases will affect the classification result of the CNN. (**a**) Small flotage, as the red circle indicates, can cling to a fish head. (**b**) Another fish’s body alongside the head region of a fish. (**c**) Reflection of a ripple in the head region. (**d**) The head shape can change because of a sharp turn of the fish.

Ideally, after applying CNN to the head feature maps contained in the segment, the segment should have only one ID. However, in practice, the segment may be assigned more than one ID. The reason is that some head feature maps may have other small flotage covering or clinging to the fish head (as illustrated in Fig. 8(a)), another fish’s body alongside the head region of fish (as illustrated in Fig. 8(b)), a reflection of a ripple (as illustrated in Fig. 8(c)), or a sharp turning of the fish (as illustrated in Fig. 8(d)). Any of these cases may cause the CNN to misclassify the head feature map as another ID. Fortunately, this situation does not last very long, so we can allow the segment be assigned multiple IDs. We use time-space continuity to link the segments according to their ID, then utilize error detection and correction to keep the segment unique in all the trajectories, and finally use trajectory verification to keep the final trajectory error free.

## Methods

### Ethics statement

All experimental procedures were in compliance with the Institutional Animal Care and Use Committee (IACUC) of Shanghai Research Center for Model Organisms (Shanghai, China) with approval ID 2010-0010, and all efforts were made to minimize suffering. This study was approved by the Institutional Animal Care and Use Committee (IACUC), and written informed consent was obtained.

### Zebrafish and environment setup

The zebrafish (*Danio rerio*) in our experiment were obtained from a local pet store. The fish are all over six months. The fish were kept in an animal facility with a 14/10 light/dark cycle, in 5-L transparent containers connected to a fish rack system with circulating water at 26.5 ± 0.5 °C and 7–7.5 pH. The fish were placed in a 20 cm × 20 cm × 15 cm transparent acrylic tank (width × length × height) with a water level of 5 cm. The water was changed the day before the experiments began. The internal walls of the fish tank were covered with white paper to eliminate reflections that could affect the tracking system. The tank was horizontally placed above an LED array panel covered with a thin diffuse film. One high-speed camera (IO Industries Canada, Flare 4M-80-CL) was mounted above the tank, with the imaging plane parallel to the water surface. The captured videos were first stored in DVR Express (IO Industries Canada, DVR Express© Core Camera Link Full, monochrome, 8 bit, Full, 1 TB) while the experiment was in process, and was exported as 8-bit bitmaps on a PC after the experiment was complete.

### The ground truth data

The ground truth data is labeled manually according to the motion coherence of zebrafish from frame to frame. Of each frame we use detection approaches to finding the head region of each zebrafish. At the beginning of each video recording, each fish is assigned a unique ID. Then we label one fish a time manually frame by frame through our labeling software (for details, see supply information and supplement video). The data labeling work is carried out by ten individuals. If a fish can not be identified after occlusion, we will go through the process of occlusion visually frame by frame to manually locate the position of fish according to the movement coherence and time context. All labeled fish trajectories will be summarized to get the most common trajectories as final ground truth.

### CNN training protocol

Our training ‘protocol’ was based on MatCovNet, a MATLAB toolbox for CNNs. The whole data only need training for 30 epochs in total, using a learning rate weight of 0.005. We wrote custom code to automate the training dataset generation without human interaction, which made optimizing the performance of the CNN much less labour-intensive. For full details of the learning parameters, see the layer parameter file in the supplementary code.

### Developed software

The software we developed for this research is available as project *fishcnntracker* on GitHub. The file *gui*_*main.m* is the entry point of the software.

## Additional Information

**How to cite this article:** XU, Z. and Cheng, X. E. Zebrafish tracking using convolutional neural networks. *Sci. Rep.* **7**, 42815; doi: 10.1038/srep42815 (2017).

**Publisher's note:** Springer Nature remains neutral with regard to jurisdictional claims in published maps and institutional affiliations.

## Supplementary Material

Supplementary Video S1

Supplementary Video S2

Supplementary Video S3

Supplementary Video S4P1

Supplementary Video S4P2

Supplementary Video S5

Supplementary Material

## Figures and Tables

**Figure 1:**
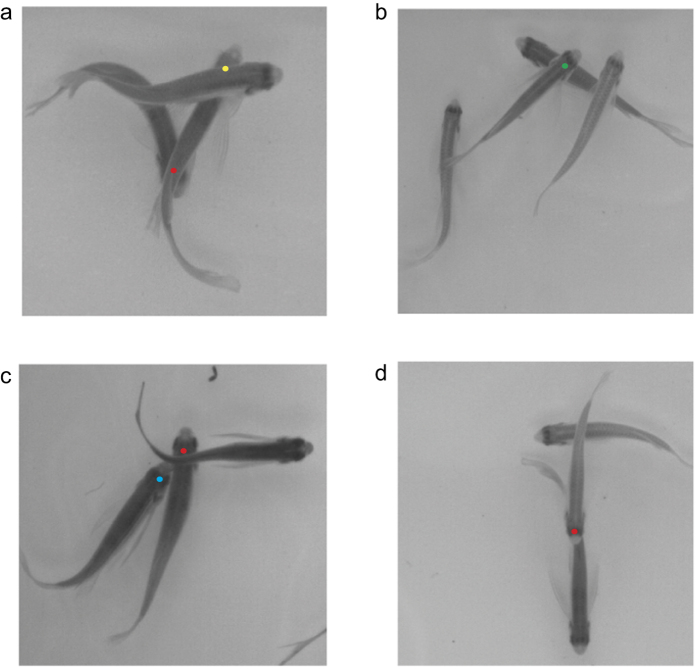
Occlusion situations. The possible ID maintains errors after occlusion, as represented by coloured dots in the images. (**a**) The IDs of red and yellow fish may be lost after occlusion or a new identity may be generated. (**b**) The ID of the green dot may be lost after occlusion or a new identity may be generated. (**c**) The ID of the blue and red dot may switch after occlusion. (**d**) The ID of the red dot may be lost after occlusion or a new identity may be generated.

**Figure 2:**
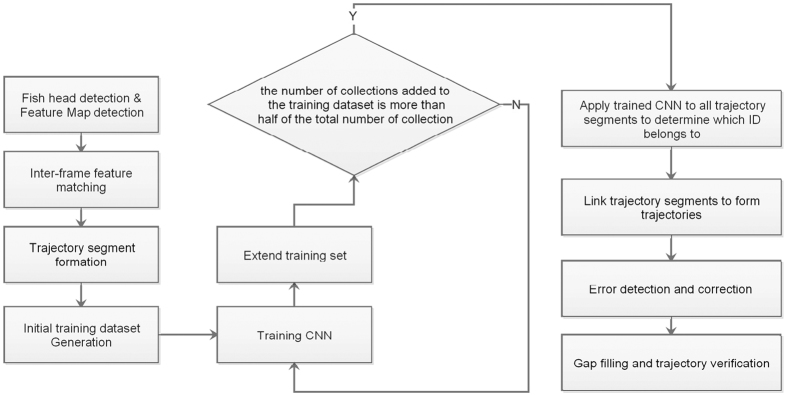
Flowchart of the tracking system.

**Table 1:** Description of the evaluation metrics.

Metric	Description
Precision	The sum of correctly tracked objects in all frames/total ground truth objects in all frames. Larger values are better.
Recall	The sum of correctly tracked objects in all frames/total tracked objects in all frames. Larger values are better.
F1-measure	The harmonic mean of precision and recall. Larger values are better.
Mostly Tracked Trajectories (MT)	Percentage of trajectories which are correctly tracked for more than 80% of their length. Larger values are better (as illustrated in Fig. 3).
Mostly Lost Trajectories (ML)	Percentage of trajectories which are correctly tracked less than 20% of their length. Smaller values are better.
Fragments (Frag)	Percentage of trajectories which are correctly tracked less than 80% but more than 20% of their length (as illustrated in Fig. 3).
ID Switch (IDS)	The frequency of identity switches after occlusion (as illustrated in Fig. 3).

**Table 2:** Evaluation of tracking performance on D1 to D5 based on the metrics in Table 2.

		Precision	Recall	F1-measure	MT	ML	Frag	IDS	Total frame number
D1		0.9831	0.9954	0.9892	1	0	0	25	2,000
D2		0.9955	0.9998	0.9976	1	0	0	6	5,000
D3		0.9967	0.9987	0.9977	1	0	0	46	15,000
D4		0.9991	0.9993	0.9992	1	0	0	27	4,000
D5	P1	0.9902	0.9910	0.9906	1	0	0	48	10,000
	P2	0.9911	0.9923	0.9917	1	0	0	42	11,000

**Table 3:** Parameter configuration of CNN network 2.

	Layer 1	Layer 2	Layer 3	Layer 4	Layer 5	Layer 6
Layer Type	Convolution	Convolution	Convolution	Convolution	Full	Full
Filter Size	3 × 3	3 × 3	3 × 3	3 × 3		
Stride	1	1	1	1		
Padding	0	0	0	0		
Channels	30	50	80	70	num × 4	num
Needs ReLU	N	N	N	Y	N	N
Needs Maxpooling	Y	Y	Y	Y	N	N
Needs Dropout	N	N	N	N	Y	N
