# Supplementary material for: Zebrafish tracking using convolutional neural networks
Source: Sci Rep. 2017 Feb 17;7:42815. doi: 10.1038/srep42815 (PMC5314376; doi:10.1038/srep42815)
Supplement: Supplementary Material [file srep42815-s7.pdf]

Zebrafish tracking using convolutional neural networks

Zhiping XU[1,*] and Xi En Cheng[2]

[1]School of Computer Science, Shanghai Key Laboratory of Intelligent Information Processing, Fudan University, Shanghai, China

[2]Jingdezhen Ceramic Institute, Jingdezhen, China

[*]zpxu@fudan.edu.cn

Additional information

S1 Video

Video sequence for D1. The video contains 14 fish swimming in the tank with a length of 2000 frames in AVI format. The resolution of the video is 2048 × 2040. The full resolution version can be found on GitHub in a repository named fishcnndata with a file named S1Video.avi.

S2 Video

Video sequence for D2. The video contains 25 fish swimming in the tank with a length of 5000 frames in AVI format. The resolution of the video is 2048 × 2040. The full resolution version can be found on GitHub in a repository named fishcnndata with a file named S2Video.avi.

S3 Video

Video sequence for D4. The video contains 11 fish swimming in the tank with a length of 4000 frames in AVI format. The resolution of the video is 2048 × 2040. The full resolution version can be found on GitHub in a repository named fishcnndata with a file named S3Video.avi.

S4 Video

Video sequence for D5. The S4 Video contains two parts recorded at different times (day 1 (P1) and day 3 (P2)) and recorded 25 wild-type zebrafish swimming in the same tank. The first part contains 10,000 frames and the second part contains 11,000 frames, both at a resolution of 2048 × 2040. The first part’ s full resolution version can be found at GitHub in repository named fishcnndata with file named S4P1Video.avi and the second part’ s full resolution version can be found at GitHub in repository named fishcnndata with file named S4P2Video.avi

S5 Video

Video sequence for instruct readers how to label their own ground truth data using our labeling software with file named S5Video.avi.

Software Prerequirements

The software can be runnable on MATLAB 2015a or above version. The prerequisites are Windows 7 or above version (or Mac OS X LION or above version, or Linux with kernel version 4.1 or above version), 16GB RAM, NVIDIA Graphic Card with compute capability above 3.0, free disk space not less than 200GB.

MATLAB Packages Required

First, go to a directory named `matconvnet\matlab` contained in this software. The system already contained Win-64 and Mac based binary-mex files with CUDA supported, so you will not bother building mex files for these two systems and run `vl_setupnn.m` directly to set the path of the MatConvNet. If you want to build mex files on your own, please refer to `http://www.vlfeat.org/matconvnet/install` for more information. Once the path has been set, please save it in the set path dialogue box (as illustrated in Fig. 1).

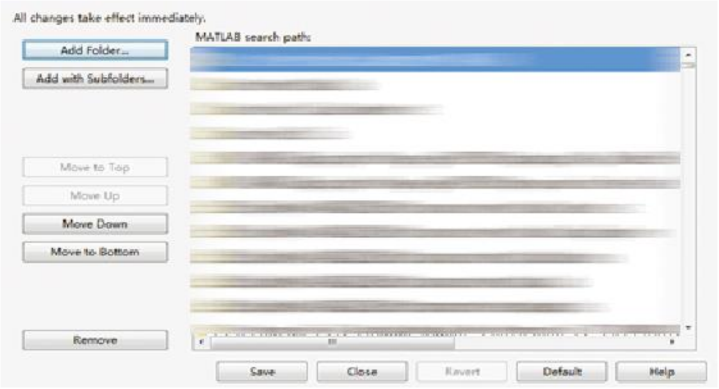

Figure 1 Set Path dialogue box.

Please refer Matlab own help system on how to Install and Use Computer Vision System Toolbox OpenCV Interface. Or just type `visionSupportPackages` and follow the install wizard.

Software

The entry point of software is `gui_main.m`, you will see following GUI.

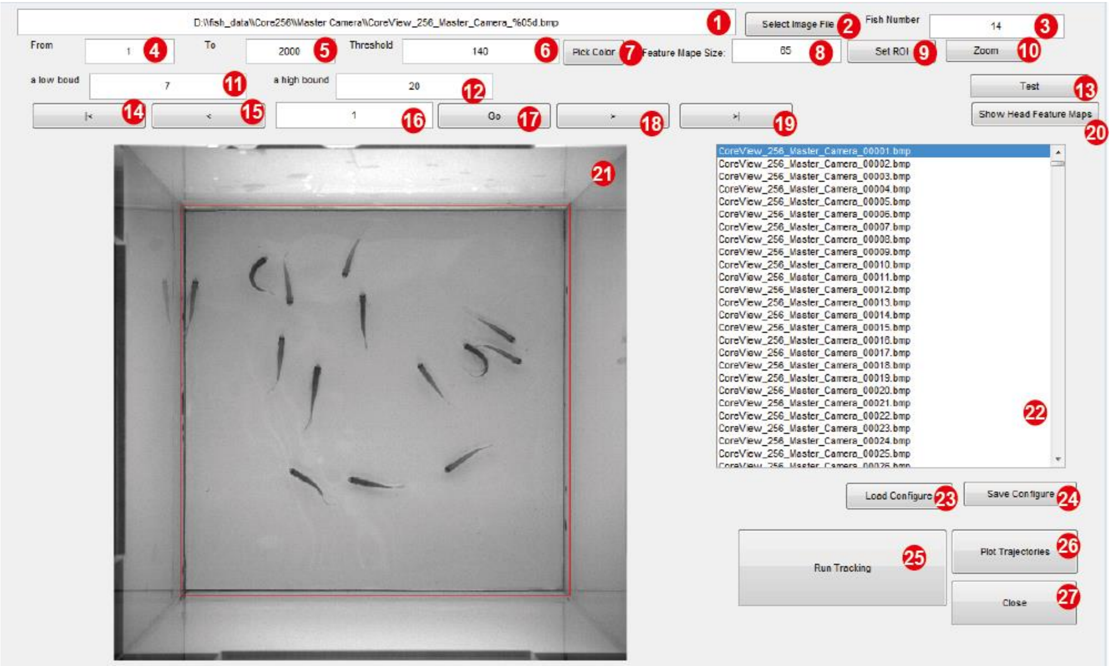

Figure 2 GUI of the tracking system

1. Filename template for processing. The area contains filename template for format string to locate the filename of the input images for next processing, please see www.mathworks.com/help/matlab/matlab_prog/formatting-strings.html for more details of writing your own format string for your own input image data.
2. The user can select an example filename to let the system to auto determine the filename template for future processing.
3. The total fish number in the experiment.
4. Start frame number of the image sequence.
5. End frame number of the image sequence.
6. Intensity threshold, which tells system ignore the pixels above given value
7. The user can use a cross hair color picker to pick intensity threshold value by right-clicking pick context menu item labeled with "Pick".
8. Feature map size determines the size of feature map, see paper section "Fish head feature map generation" to get more information.
9. Set region of interest of input image.
10. Zoom the image view to get a clearer view of the details in the image.
11. The lower boundary of the specified scale range, see paper section "Fish head feature map generation" to get more information.
12. The upper boundary of the specified scale range, see paper section "Fish head feature map generation" to get more information.
13. Test if can correct detect fish and obtain correct feature map of fish. If the system can successfully detect fish head, the center of the head region will show a green circle as illustrated in Fig 3.
14. Go to the first frame of the image sequence.
15. Go to the previous frame according to the current frame.
16. The indicator of the current frame number can be manually entering desired frame number and push "Go" button to go to the specified frame.

17. Go to frame specified in the indicator of current frame number.
18. Go to next frame according to the current frame.
19. Go to the last frame of the image sequence.
20. Show head feature maps and silhouettes of detected fish.
21. The view of the current frame.
22. The filename list of current working directory.
23. Load parameter configures file saved in the previous session.
24. Save parameters show in the GUI to the parameter configure file.
25. Run tracking system.
26. After tracking process is done, the user can press this bottom to draw trajectories of all fish and single trajectory of each fish and can produce video file contained trajectories of each or all fish.
27. Close the GUI.

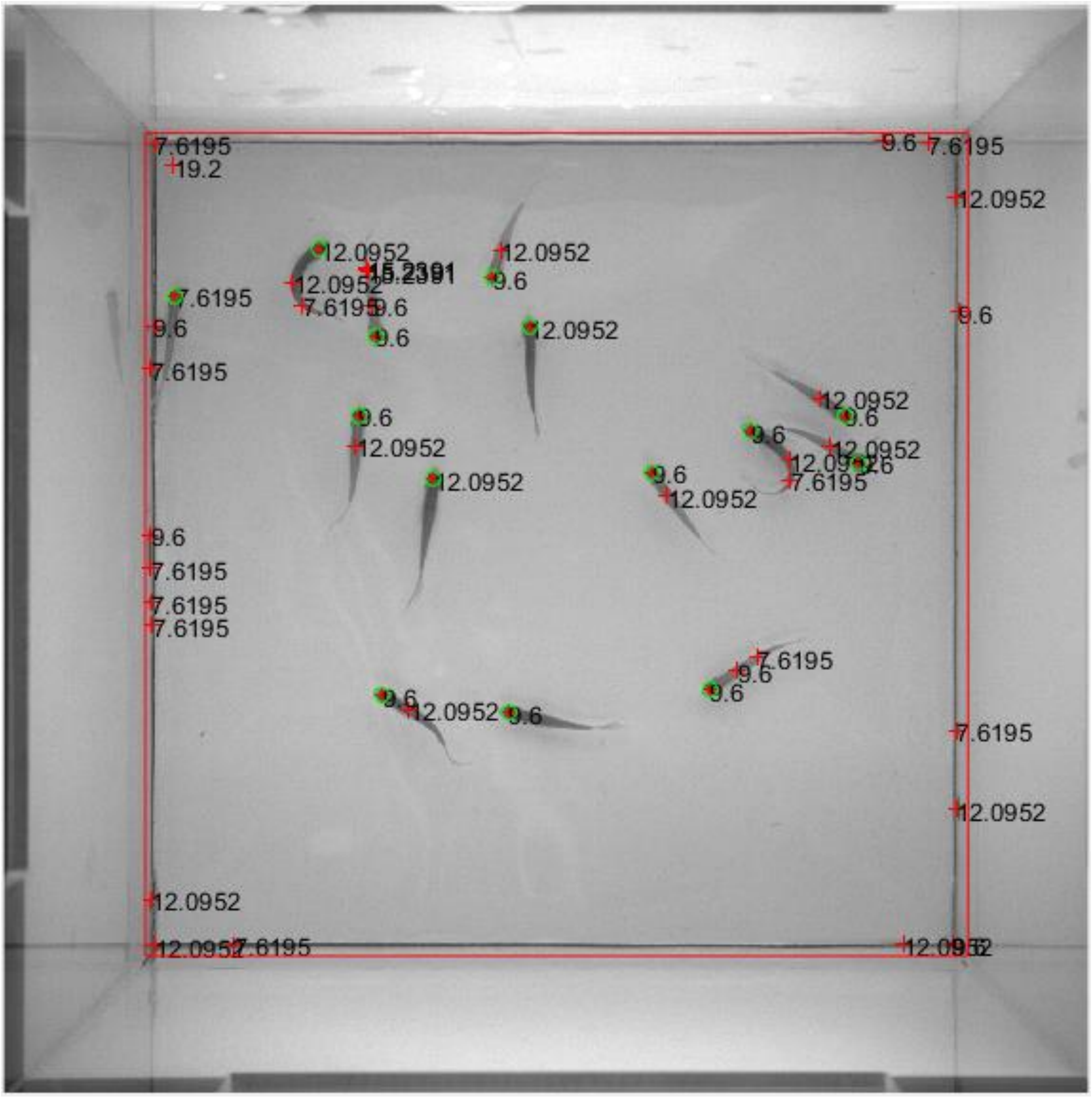

Figure 3 Successfully detected fish head. If fish head can be successfully detected, the green circle marks indicate the detected fish head will be shown, and the red cross mark indicates the candidate fish head, the number beside the red cross mark indicates the scale size of the detected point, which will be filtered by 11 and 12 parts in the Fig. 2

Ground truth data labeling software

The ground truth data labeling process contains two phases: 1) label linked segments of each fish; 2) manually fill the gap between the linked segments to form full length of ground truth trajectories.

Phase I

When the user enters 'gui_mark_learning', the following GUI will bring up. It will begin the first stage of ground truth data labeling.

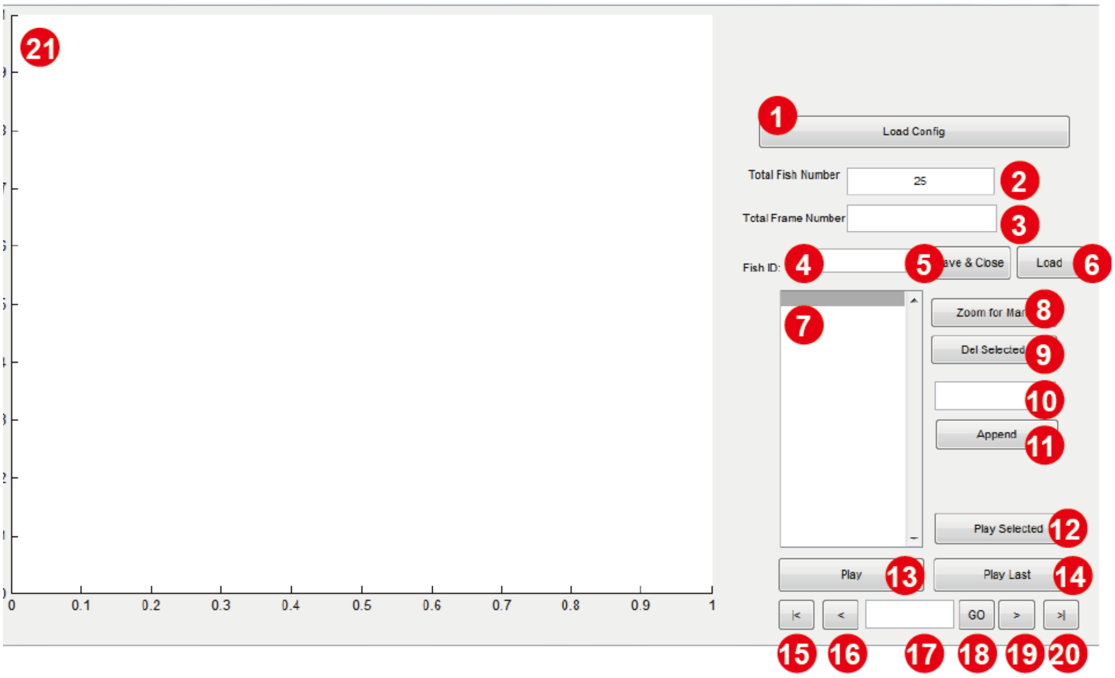

Figure 4 GUI of ground truth labeling software (Phase I)

1. Load configuration generated by `gui_main.m`, once the configuration is loaded, this button will turn into green like this, 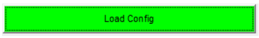

meanwhile, it will show the fish number in 2 and total frame number to be processed in 3.
2. The total fish number in the experiment.
3. Total frame number in the experiment.
4. Fish ID selector, the user can choose fish ID to start data labeling.
5. Save current labeling data for next phase and close window.
6. Load previous labeling data.
7. The linked trajectory segments list of current selected fish ID in 4.
8. This button will zoom current frame in 21, and let the user choose which segment will be linked to the current segment.
9. Delete selected segment in 7.
10. The user can manually enter segment ID number here and press 11 to append this ID to the linked trajectory segments list in 7.
11. Append segment ID number in 10 to the linked trajectory segments list in 7
12. Play animation of selected segment of the selected fish in 21.

13. Play animation of whole linked trajectory segments list of current selected fish ID in 21
14. Play animation of last linked trajectory segments list of current selected fish ID in 21
15. Go to the first frame of the image sequence.
16. Go to the previous frame according to the current frame.
17. The indicator of the current frame number can be manually entering desired frame number and push “Go” button to go to the specified frame.
18. Go to frame specified in the indicator of current frame number.
19. Go to next frame according to the current frame.
20. Go to the last frame of the image sequence.
21. The view of the current frame.

The process of labeling linked segments

1. The user should press “Load Config” button to load the configuration into the system. When the button turns into green, the system is ready for ground truth data labeling.
2. Choose a fish to be labeled.
3. After the fish is selected, the system will bring up all linked segment IDs of related fish. The user can press “Play Last” button to play the animation of current fish, the trajectory of current fish in current segment is shown in red line, the candidate linked segments are shown in green line. At beginning of each line, there is a blue label in the format “<ID#>@<Dif>”, “<ID#>” indicates the segment number and “<Dif>” is the frame number difference between current frame and the beginning part of the segment. When the following like situation is shown in current view (see Fig. 5).
4. The user can press “Zoom for mark” button to select net link segment, then the mouse

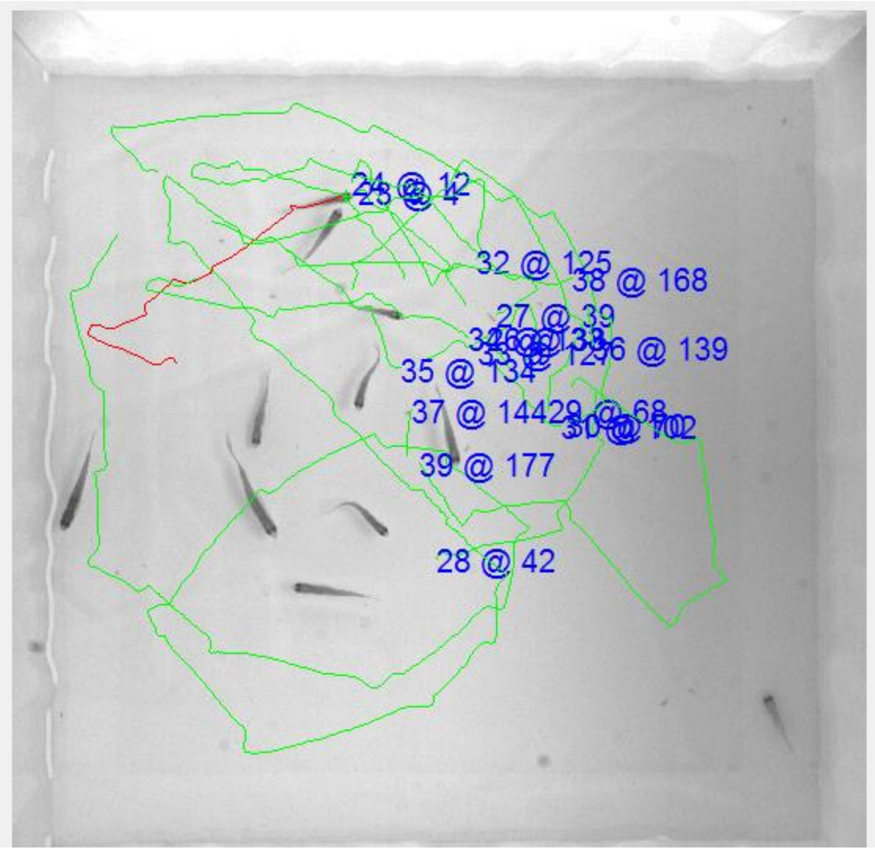

Figure 5 The status of view.

pointer is changed into a magnifier, which user can use a wheel on the mouse to zoom in or out in the view or click a spot in the view to zoom specified region.

5. The user then can continuous press ">" button to go to next frame and watch current fish head position, when current fish head position is at the beginning of the segment and the segment label is changed like "<ID#>@0" (see Fig. 6), then the user can click right mouse button near the segment label, and choose "Mark" to add this segment to the current link segment list.

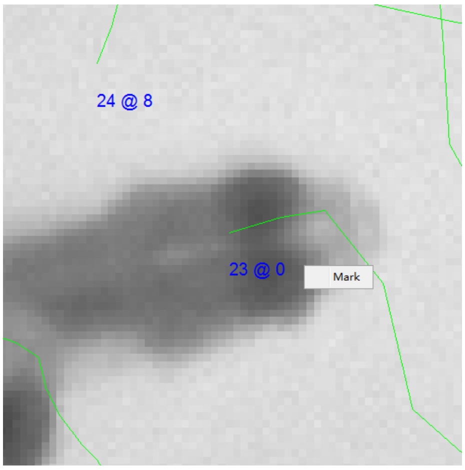

Figure 6 Add segment to linked segment list

6. The user can press "Play Last" button to play the animation of last added segment of current fish, the system will stop at the end of the segment, then a view like Fig.5 will show up, then the user could repeat step 4 to 6 until the whole sequence is processed. Then the user can select another fish to start this process again until all fish have been labeled. When all fish have been labeled, the user can press "Save & Close" button to save labeled data for phase II.

Phase II

When the user enters 'gui_mark_groundtruth.m', the following GUI will bring up. It will begin the second stage of ground truth data labeling.

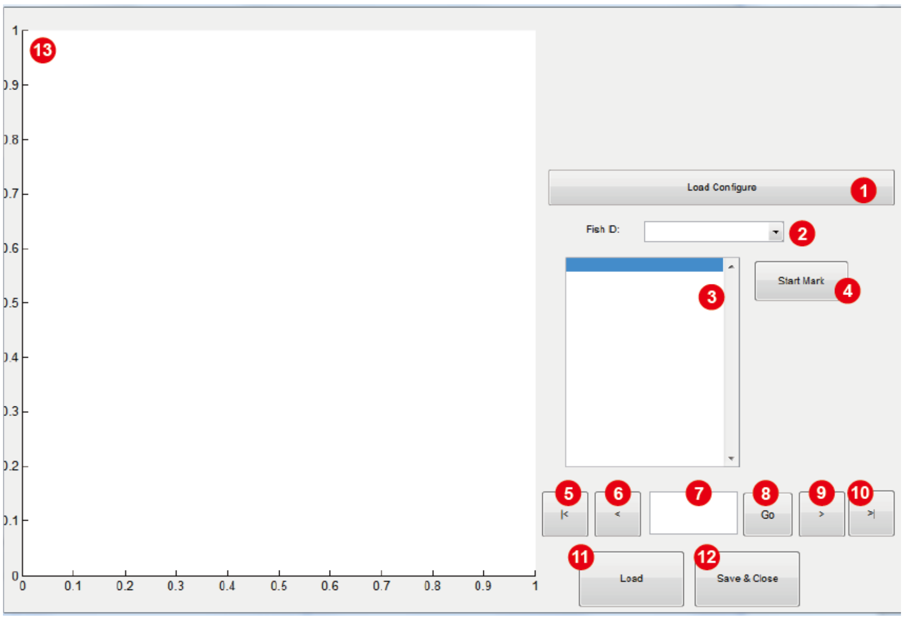

Figure 7 GUI of ground truth labeling software (Phase II)

1. Load configuration generated by `gui_main.m` and data saved in phase one into the system, once the data are loaded, this button will turn into green like this. 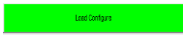 And fish ID list in 2 will be filled with alll fish IDs in the experiment.
2. Current Fish ID selector. When a Fish ID is selected, its labeled linked segment ID list willl be shown in 3.
3. The linked segment ID List of current fish ID.
4. Start manually mark head position between stwo linked segments.
5. Go to the first frame of the image sequence.
6. Go to the previous frame according to the current frame.
7. The indicator of the current frame number can be manually entering desired frame number and push "Go" button to go to the specified frame.
8. Go to frame specified in the indicator of current frame number.
9. Go to next frame according to the current frame.
10. Go to the last frame of the image sequence.
11. Load previous labeling data.
12. Save labled trajectories as ground truth.

The process of labeling the missing trajectories in the gaps between linked segments

1. The user should press "Load Configure" button to load the configuration and previous labeled linked segment data into the system. When the button turns into green, the system is ready for ground truth data labeling.
2. Choose a fish to be labeled. After a fish is selected, its related linked segment IDs will be shown in the segment ID list.
3. Select segment ID in in list, and press "Start Mark" button, this will let the user start mark the missing trajectories between the selected segment and the next linked segment, for example, if the user want to fill the gap between segment ID 1 and 23, then the user need to select segment with ID 1.

4. After "Start Mark" button is pressed, then the mouse pointer is changed into a magnifier, which user can use a wheel on the mouse to zoom in or out in the view or click a spot in the view to zoom specified region. Meanwhile, the following like situation will show in view as illustrated in Fig. 8.

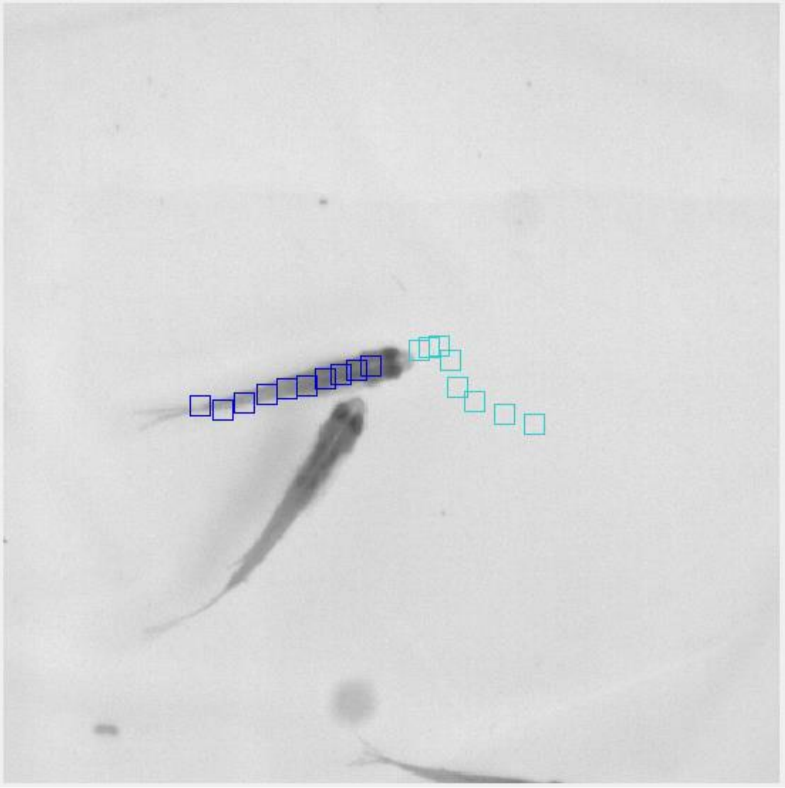

Figure 8 The gap filling status view

As illustrated in Fig. 8, the dark blue squares indicate previous trajectories of current fish and cyan squares indicate following-up trajectories of current fish, the user can click right mouse button at the head center of the fish and select "Mark" context menu item to mark the position of fish head in current frame, then the system will automatically jump to next frame waiting for next mark. This process can be stopped when an orange cross hair is shown in the view (as illustrated in Fig. 9), it means current frame is just same as the beginning frame of the next linked segment.

5. The user can choose another segment to fill the trajectory gaps, then the user could repeat step 3 to 4 until the whole sequence is processed. Then the user can select another fish to start this process again until all fish have been labeled. When all fish have been labeled, the user can press "Save & Close" button to save the final ground truth data.
6. When "Save & Close" button is pressed, a dialogue will show up to let the user input the filename of ground truth data.

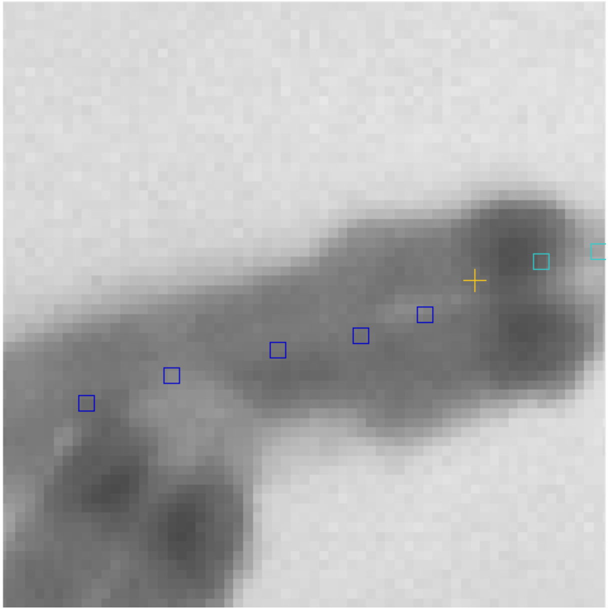

Figure 9 The current frame is same as the beginning frame of the next linked segment
